# Adapting State-of-the-Art Deep Language Models to Clinical Information Extraction Systems: Potentials, Challenges, and Solutions

**DOI:** 10.2196/11499

**Published:** 2019-04-25

**Authors:** Liyuan Zhou, Hanna Suominen, Tom Gedeon

**Affiliations:** 1 Research School of Computer Science College of Engineering and Computer Science The Australian National University Canberra Australia; 2 Machine Learning Group Data61 Commonwealth Scientific and Industrial Research Organisation Canberra Australia; 3 Faculty of Science and Technology University of Canberra Canberra Australia; 4 Department of Future Technologies Faculty of Science and Engineering University of Turku Turku Finland

**Keywords:** computer systems, artificial intelligence, deep learning, information storage and retrieval, medical informatics, nursing records, patient handoff

## Abstract

**Background:**

Deep learning (DL) has been widely used to solve problems with success in speech recognition, visual object recognition, and object detection for drug discovery and genomics. Natural language processing has achieved noticeable progress in artificial intelligence. This gives an opportunity to improve on the accuracy and human-computer interaction of clinical informatics. However, due to difference of vocabularies and context between a clinical environment and generic English, transplanting language models directly from up-to-date methods to real-world health care settings is not always satisfactory. Moreover, the legal restriction on using privacy-sensitive patient records hinders the progress in applying machine learning (ML) to clinical language processing.

**Objective:**

The aim of this study was to investigate 2 ways to adapt state-of-the-art language models to extracting patient information from free-form clinical narratives to populate a handover form at a nursing shift change automatically for proofing and revising by hand: first, by using domain-specific word representations and second, by using transfer learning models to adapt knowledge from general to clinical English. We have described the practical problem, composed it as an ML task known as information extraction, proposed methods for solving the task, and evaluated their performance.

**Methods:**

First, word representations trained from different domains served as the input of a DL system for information extraction. Second, the transfer learning model was applied as a way to adapt the knowledge learned from general text sources to the task domain. The goal was to gain improvements in the extraction performance, especially for the classes that were topically related but did not have a sufficient amount of model solutions available for ML directly from the target domain. A total of 3 independent datasets were generated for this task, and they were used as the training (101 patient reports), validation (100 patient reports), and test (100 patient reports) sets in our experiments.

**Results:**

Our system is now the state-of-the-art in this task. Domain-specific word representations improved the macroaveraged F1 by 3.4%. Transferring the knowledge from general English corpora to the task-specific domain contributed a further 7.1% improvement. The best performance in populating the handover form with 37 headings was the macroaveraged F1 of 41.6% and F1 of 81.1% for filtering out irrelevant information. Performance differences between this system and its baseline were statistically significant (*P*<.001; Wilcoxon test).

**Conclusions:**

To our knowledge, our study is the first attempt to transfer models from general deep models to specific tasks in health care and gain a significant improvement. As transfer learning shows its advantage over other methods, especially on classes with a limited amount of training data, less experts’ time is needed to annotate data for ML, which may enable good results even in resource-poor domains.

## Introduction

### Background

Machine learning (ML) is being studied and used in a variety of health informatics applications (eg, disease progression prediction, therapy planning, medical diagnostic reasoning, and automatic patient management) as a way to help clinical experts to improve the efficiency and quality of medical care [1,2].

A clear majority of these applications use supervised learning, which infers knowledge from labeled training data. However, because of stringent restrictions on the use of clinical data [3], data collections on real health care scenarios that are open for research and development are very limited [4]. Moreover, the few available sources have limitations such as research-only use [5], nondisclosure of data [6], or limited commercial licenses [7].

Zheng et al [8] proposed an information extraction (IE) framework called IDEAL-X, which uses online learning techniques to update the learning model based on user feedback. Although the performance of their system looks very impressive, the types of text this system is able to extract are limited to 5, and these types such as age, gender, and medicine can be easily retrieved with rule-based systems rather than ML systems. Leroy introduced a rule-based automated IE system that extracts diagnostic criteria from electronic health records for autism spectrum disorders [9]. As the rules are manually generated based on human observations of 1 specific data set, their system cannot be generalized to other tasks.

**Figure 1:**
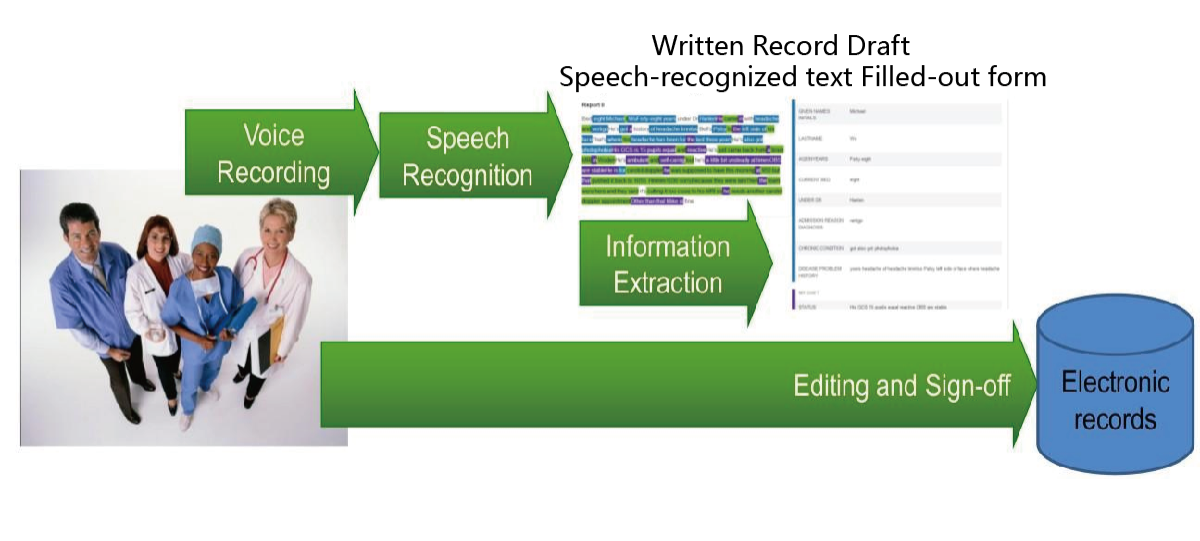
A processing pipeline that transforms verbal clinical handover information into electronic structured records automatically.

In our previous study [4], we have already (1) discussed the importance of comprehensive record keeping along with information flow in health care in general and clinical handover in particular, (2) developed and freely released a set of 101 synthetic clinical handover cases with verbatim conversations and associated audio recordings constructed by a nurse with over 12 years of experience in clinical practice to make sure the cases are closely matched with the typical data found in a nursing shift change, and (3) introduced and evaluated a cascaded system that uses speech recognition (SR) to recognize verbal clinical handover information and IE to fill in a handover form for clinical proofing and sign-off (Figure 1).

### Objectives

In this study, we have released another 2 datasets that follow exactly the same format as our first release to supplement the original dataset called the National Information and Communications Technology Australia (NICTA) Synthetic Nursing Handover Data. These 3 independent datasets target researchers who are training, validating, and testing ML-based SR and IE methods for the handover record-keeping task. A description of our dataset is available in Multimedia Appendix 1.

More importantly, in this study, we have improved our IE performance by using an ML method, which learns from other data collections and transfers this knowledge to the handover task. Processing correctness is crucial in medical informatics applications; our benchmark results show that this task is very challenging [4], and the previous state-of-the-art result on this task was only 38.2% on macro F1 [10]. Even with our supplementary data, the size of the in-domain training set is still not adequate to train a traditional multilayer neural network (NN) model for our IE task composed as a 50-class classification.

Generating or getting access to a large manually labeled training corpus for this task is not easy. Fortunately, distributed word representations, which can be learned from unlabeled data, have recently been shown to have high utility in many natural language processing (NLP) applications [11-14]. In this study, we have investigated whether pretrained word embeddings generated from general Web text could improve our system performance in the IE task, even though it is relatively domain-specific. Furthermore, we are also interested to discover if training supplementary word embeddings based on a domain-related corpus is more helpful than from a general English corpus.

Transferring the knowledge learned from another domain to our task is another way to cope with the problem of lack of training instances. This method has shown its effectiveness in previous studies [15,16]. In this study, we have implemented a transfer learning–based approach to adapt weights of features and labels from different source data corpora to gain an improvement in the clinical handover IE task. More specifically, if we define our current task as the target domain, then the dataset that we want to adapt weights from is the source domain, so we first train sequence labeling models on a source domain training corpus and then learn the correlations between source labels and the labels in our task. After this, we use the model parameters of each related class in the source model to initialize our conditional random field (CRF) model in our clinical handover IE task. To extend the study, we have also explored whether models learned from a source corpus, which is close to a clinical domain, are more helpful than models trained on a generic, large labeled corpus.

To summarize the contributions of this study, we have released the data to study SR and IE and introduced a state-of-the-art IE method for the handover task. The method is based on transfer learning and compares with both the most recent deep learning (DL) approaches and more traditional CRFs for sequence labeling.

## Methods

### NICTA Synthetic Nursing Handover Data

To fulfil the purpose of constructing systems to automatically generate structuring of the narrative documents from nursing shift change speech and handover, NICTA Synthetic Nursing Handover Data [17-19] was created at NICTA/Data61 from 2012 to 2016. Their main author was a professional Registered Nurse (RN), Maricel Angel, who has over 12 years of experience in clinical nursing, based on general practice in medical wards. Therefore, the text is very similar to real documents in typical clinical scenarios.

**Figure 2:**
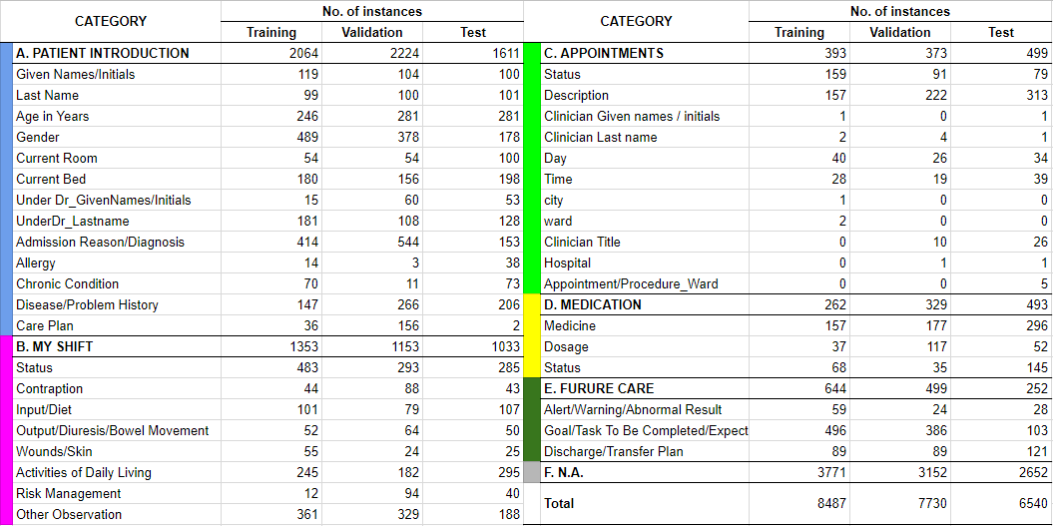
Descriptive statistics of text snippets highlighted in the training, validation, and test set.

This data collection of 301 records in total contained 3 disjoint subsets for training (101 records), validation (100 records), and testing (100 records; Figure 2). All 3 subsets were created under a consistent practice with the same standards as used by Suominen et al [4]. Each record contains a patient profile; a written, free-form clinical handover for this profile; a voice speech record of the handover; and, finally, a written, structured document. To represent the most common chronic diseases and national health priority areas in Australia [20], 4 kinds of patients (ie, cardiovascular, neurological, renal, and respiratory patients) were introduced into each subset and independently followed a uniform distribution to provide a balanced demographic sample. The structured document includes annotation of 5 classes (PATIENT INTRODUCTION, MY SHIFT, APPOINTMENTS, MEDICATION, and FUTURE CARE), which were further divided into 37 subclasses, supplemented by the category of not applicable (NA) for irrelevant information.

### Word Representation

Word embedding is a vector matrix learned from an unlabeled text corpus that maps vocabulary to a dense vector space. It attempts to model the distributional hypothesis that words that occur in similar contexts tend to be semantically similar. It has been shown to contribute to a variety of NLP tasks even without using any other features [21].

To capture word vector representations from large amounts of unlabeled text, we adapted a skip-gram model [22] that uses each current word to predict words in the neighboring context. The training objective of the skip-gram model is to maximize the averaged log probability over all training cases (*T*) of appearance of the context word *w_{t+j}* given the current word *w_t*, where *j* is the offset of the context word from the current word in a context window size of *c*:

1/T∑_{T}^{t=1}(∑_{-c≤j≤c,j≠0}(log p(w_{t+j}|w_t)))

Then, applies softmax to each context word *w_O* of a given occurrence of word *w_I*:

P(w_O|w_I)=exp(v’^{T}_{w_O}v_{w_I})/∑^W_{w=1} exp(v’^T_w v_{w_I})

...where v_w is the input and v’_w is the output word embedding of a word *w*, and *W* is the size of the training vocabulary.

Out of the 2 variations to optimize computational efficiency of the skip-gram model, we have used negative sampling rather than hierarchical softmax because, for sequence tagging tasks in NLP, it can maintain more semantic information during the training process [23] and obtain better results [24]. Rather than calculating *exp(v’^T_w v_{w_I})* for all *w* in the vocabulary when *calculating log p(w_{t+j}|w_t)*, negative sampling replaces it with a logistic regression and distinguishes a context word *w_O* from noise (negative samples):

log σ(v’^T_{w_O} v_{w_I}) + ∑^k_{i=1} E_{w_i~U(w)} [log σ(-v’^T_{w_i} v_{w_I})],

...where *k* is the amount of negative samples for each data sample, and *U(w)* is a unigram distribution of words.

To integrate context information from a new domain-specific corpus *D_1* (eg, our clinical handover dataset), we need to update our existing word embeddings that were learned from a general large text collection *D_0*. However, this comes with a significant challenge in that the original word embeddings were trained on a very large corpus whereas our target domain training data are normally much smaller in size. More specifically, if we compare the vocabulary between *D_0* and *D_1*, *V_0 ∩ V_1* is the intersection between 2 vocabularies, which is mainly composed of more general terms compared with *V_1/V_0*: the relative complement of *V_0* with regard to *V_1*. As word vectors in *V_0* have already been trained for several epochs and converged to our desired values, we do not want to change them significantly. However, new words that were just introduced to the vocabulary from *V_1/V_0* contain domain-specific, related terms about which we care the most. Owing to the limitations in the available amount of training samples, a large learning rate at the beginning is useful to adjust these vectors to the regions they belong. Using the original skip-gram algorithm will potentially either adjust the already converged vectors from *V_0* away from their optimized values or the new word vectors will not get close to words that are similar to them in the vector space. To cope with this problem, we used 2 strategies in our experiments: averaged initialization as well as different learning rates.

In averaged initialization, our assumption was that words in similar contexts would have similar meanings or have grammatical similarities. Although this is not always true, this strategy helps in learning the new words, as they start from an (averaged) optimized point rather than from scratch. To model this, whenever a new word appears, the initial value of the vector is set to be the averaged value in each dimension of words in the same sentence:

1/S∑^{S}_{i=1}
**v**_i, where
**v**_i=
**v**, if
**v**∊ V_0,

=
**v**_0, otherwise

...where *S* is the sentence length and **v***_0* denotes the vector in *V_0*, which represents the vector of *unknown* words.

During the training procedure, we have also used different learning rates for words in *V_1/V_0* in contrast with *V_0∩V_1*:

α_t=α_{0_new}(1–t/n), t≠0, and
**v**∊V_1/V_0

α_t=0.2, t=0, and
**v**∊V_1/V_0

α_t=α_{0_old}(1–t/n), t≠0, and
**v**∊V_1∩V_0

α_t=α_{0_new}(|V_1/V_0|/|V_0∪V_1|), t≠0, and
**v**∊V_1/V_0

...where *n* is the amount of samples input to the network. For new words, the initial learning rate *α_{0_new}* is set to 0.2 and decreases over time. For words that are already in *V_0*, the initial learning rate is set to the portion of new words in the entire vocabulary: the more learning samples we have for new words, the larger the initial learning rate for old words.

### Transfer Learning for Sequence Labeling

For sequence tagging tasks that use supervised ML, the amount and purity of training data is crucial to the performance of our system. As the complexity of learning a 40-class classifier is high, for some labels, there is only 1 case in the training set, which could not be generalized to infer good functions [25]. Therefore, introducing more training data could improve the final results. However, when more training instances are not available, or are extremely costly in human labor, transfer learning, which adapts weight matrixes from functions trained with another dataset and applied to the current task, is another way to gain knowledge of labels with limited training instances.

**Figure 3:**
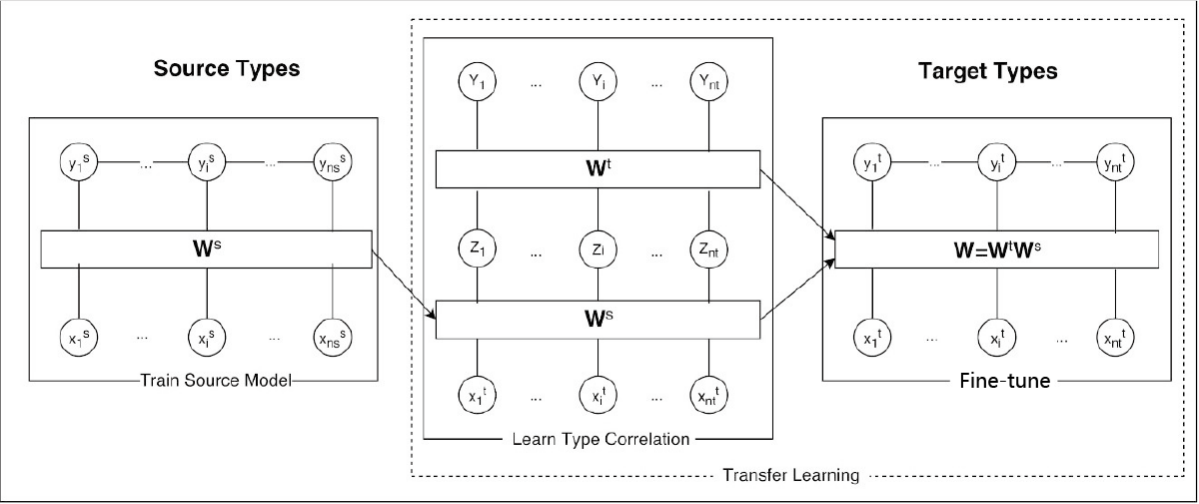
Transfer learning model structure.

The underlying idea of transfer learning is simple: in deep NNs, there are several hidden layers between the input and output layers; as data feed forward from the input layer to the output layer, the composition of features is learned from earlier layers [26]. A typical sequence tagging structure can be demonstrated as the left block in Figure 3: neurons in lower layers tend to capture some common, nondomain, or task-specific concepts, and later layers would concatenate these features and generate higher-level concepts. Therefore, weights learned from other datasets or even other tasks can be potentially reused as long as the structure of the later layers are consistent between the source model and target model.

Several strategies of transfer learning on different NLP tasks and domains have been explored. A simple strategy is to copy all weight matrixes in the source model to the target model and fine-tune the target model with new data [27], which successfully outperformed the leading team in the Multilingual Emoji Prediction task [28] by 1.55% without any feature engineering procedure. However, this method requires the source and target model to have the exact same structure. An alternative strategy is to map annotations from different datasets into 3 consistent labels and use source domain model parameters directly as initializations for a target domain model in named entity recognition [16]. Finally, human adjustment of rules and features [29,30] or clustering labels from source domain and target domains to automatically generate label mappings from one dataset to the other [31] can be applied as transfer learning strategies. However, their productivity may be limited when adapting a general source model to multiple tasks and also the generated mappings might not be satisfactory, especially when 2 datasets have dramatic differences in terms of phraseology and grammar.

The method we have introduced in our study was able to transfer knowledge to a target domain that does not match labels in the source domain, does not depend on human integration during the label mapping process, and is able to map labels from very different datasets. This method follows 3 steps (Figure 3): the first step trains a CRF model on the source domain, the second step uses the weight matrix of the source model *W^s* to train a 2-layer CRF that predicts a target domain label given a source domain label, and, finally, the third step is to initialize the parameters of the target domain model using the product of *W_s* and the second layer weight matrix *W^t* obtained in step 2.

First, in the source model training step, a linear-chain CRF model is trained on a large labeled source dataset. For each word *x_i* in a sequence **x**, *y_i* ∊ Y is the label of *x_i*, where Y is the label set. Then (**x**; **y**) is a sequence of word label pairs; a linear chain CRF is a distribution *p* (**y**| **x**) that takes the form:

p(
**y**|
**x**) = 1/Z ∏^{L}_{l=1} (
**W**^f f(y_l,
**x**) +
**W**^g g(y_{l-1}, y_l))

...where *Z* is a normalization constant, *L* is the length of **x**, *f(y_l*, **x***)* is a real value feature function of **x**, *g(y_{l-1}, y_l)* is a feature function of current label *y_l* and previous label *y_{l-1}* in the sequence to capture the cooccurrence between adjunct labels, and contains the parameters of the feature functions.

For now, the source CRF model can be seen as an NN with 2 hidden layers: the lower layer, which is connected by **W**^*f*, and the upper layer connected by **W**^*g* (Figure 3). However, because the information that is captured by **W**^*g* is normally domain- and task-specific, label correlations do not always have similarities between different domains, which would thus not be suitable to be transferred to the other task. In contrast, the lower layer learned correlations between words and source labels, which is what we are interested in, are actually a logistic regression model:

σ(y*,
**x**_i,
**W**^f) = exp(
**W**^f_{.y*}f(y*_i,
**x**_i)) / ∑_{y∊Y}exp(
**W**^f_{.y}f(y,
**x**_i))

Second, in the source label and target label correlations step, we have propagated the label probabilities from the later layer of the source domain model to another logistic regression classifier to form a 2-layer linear-chain CRF model that predicts target domain labels and uses source domain labels to learn correlations between the source and target labels. More specifically, the linear layer from the source model can be defined as:


**a**_i=
**W**^s f(y,
**x**_i)

...where each **a**_*i* is the probability for each source label and **W**^*s* denotes the weight matrix from source domain. After this layer, a linear regression classifier takes the output from **a**_*i* to predict target labels:

p(y’|
**a**)=σ(y’,
**a**_i;
**W**^t),

...where *y’* is the target type. This is equal to:

p(y’|
**x**)=σ(y’,
**x**_i;
**W**^t
**W**^s)

After **W**^*s* and **W**^*t* are trained, we were able to initialize a CRF model to predict target labels with **W**^*f* = **W**^*t* **W**^*s* as the third step:

p(
**y**|
**x**)=1/Z ∏^{L}_{l=1} (
**W**^t
**W**^s f(y_l,
**x**))

During this procedure, the parameters of label NA, **W**^*t_{NA}* are reset to be zeros because the amount of instances of NA in the text corpus is much larger than that of other labels, which would cause the model to be biased to the dominant class.

Theoretically, the parameters of feature functions will converge to the same weights with a random initialization model when the number of iterations is large enough because the loss function is convex. However, our aim was to inherit knowledge from the source domain, updating **W**^*t* **W**^*s* too often would cause the model to forget what it has learned so far. Therefore, early stopping and adaptive gradient algorithm (AdaGrad) [32] are applied to preserve the learned source domain knowledge.

### Performance Evaluation and Experimental Design

To compare the performance of systems, we have measured the precision, recall, and F1 (harmonic mean of precision and recall) over all categories [33]. More specifically, microaveraged F1 and macroaveraged F1 are calculated. As our purpose was to emphasize on systems that perform well in all classes rather than only in the classes that have majority instances, macroaveraged F1 was selected as the main evaluation measurement.

The resources used in our experiments were derived from 7 different corpora (Table 1). Among them, 3 were general English text based, 3 were specific to the English health care domain, and 1 was the test set. Details of the test set are available in Multimedia Appendix 2.

1. General English Corpora:
  - *English Wikipedia* is a freely available corpus from the September 2014 version of all pages from all Wikipedia wikis. It contains more than 3 million English pages, 100 million sentences, and 3.4 billion words in total after cleaning.
  - *University of Maryland, Baltimore County (UMBC) WebBase corpus* is a dataset containing a collection of 100 million English Web pages from more than 50,000 websites with over 3 billion words processed from the February 2007 crawl by the Stanford WebBase project [34].
  - *One Billion Words Benchmark for Language Modeling* is a freely available standard corpus of 4.2 GB (0.8 billion words) for building and testing language models [35].
2. Medical Domain–specific English Corpora:
  - *I2B2* is a collection of fully deidentified clinical records provided by the I2B2 National Centre for Biomedical Computing funded by U54LM008748 and was originally prepared for *Shared Tasks for Challenges in NLP for Clinical Data* organized by Uzuner, I2B2, and SUNY [36-39].
  - *PubMed* is a free resource containing over 27 million citations to the biomedical literature and publication abstracts derived from MEDLINE, life science journals, and Web books. It was developed and is maintained by the National Centre for Biotechnology Information at the US National Library of Medicine (NLM).
  - *PubMed Central (PMC) Open Access Subset* contains over 1 million biomedical articles from PMC, which is a free archive of biomedical and life sciences journal publications at the US National Institutes of Health's NLM.
  - *NICTA TRAIN* is the NICTA Synthetic nursing handover dataset, an open clinical dataset of 3 sets of nursing handover records, very similar to real documents in Australian English. Each record consists of a patient profile, spoken free-form text document, written free-form text document, and written structured document [40].

**Table 1:** Word embeddings training corpora.

Corpus	Size	Source
English Wikipedia	3.4 billion words	Wikimedia downloads [41]
UMBC^a^	>3 billion words	UMBC WebBase corpus [42]
One Billion	0.8 billion words	One Billion Word Benchmark for Measuring Progress in Statistical Language Modeling [43]
I2B2	18,082 unique words	I2B2 NLP^b^ research data sets [6]
PubMed	27 million records	PubMed resources [44]
PubMed Central	1 million articles	PubMed resources [45]
National Information and Communications Technology Australia Train	101 records	Hospital handover forms [17]

^a^UMBC: University of Maryland, Baltimore County.

^b^NLP: natural language processing.

**Table 2:** Mapping of entity types between the source and target corpora.

In-domain source: Bolt, Beranek and Newman	Out-domain source: I2B2	Target: NICTA^a^ Clinical Handover
PERSON	PATIENT	PATIENT_INTRODUCTION/Given_names
PERSON	PATIENT	PATIENT_INTRODUCTION/Last_name
PERSON	DOCTOR	PATIENT_INTRODUCTION/Under_Dr:Given_names
PERSON	DOCTOR	PATIENT_INTRODUCTION/Under_Dr:Last_name
PERSON	DOCTOR	APPOINTMENTS/Clinician:Last_name
PERSON	DOCTOR	APPOINTMENTS/Clinician: Given_names
ORGANIZATION:HOSPITAL	HOSPITAL	APPOINTMENTS/Hospital
DATE:AGE	—^b^	PATIENT_INTRODUCTION/Age_in_years
PER_DESC	—	PATIENT_INTRODUCTION/Gender
PER_DESC	—	APPOINTMENTS/Clinician Title
GPE:CITY	LOCATION	APPOINTMENTS/City
DATE:DATE	DATE	APPOINTMENTS/Day
TIME	—	APPOINTMENTS/Time
CARDINAL	ID	PATIENT_INTRODUCTION/Current_room
CARDINAL	ID	PATIENT_INTRODUCTION/Current_bed
PRODUCT:OTHER	—	Medication/Medicine
SUBSTANCE:DRUG	—	Medication/Medicine
QUANTITY:3D (volume)	—	Medication/Dosage
QUANTITY:OTHER	—	Medication/Dosage
QUANTITY:TEMPERATURE	—	My_shift/Status
QUANTITY:WEIGHT	—	My_shift/Status
SUBSTANCE:FOOD	—	My_shift/Input_diet
FACILITY	—	APPOINTMENTS/Ward
DISEASE	—	PATIENT_INTRODUCTION/Admission_reason/diagnosis
DISEASE	—	PATIENT_INTRODUCTION/Chronic_condition
DISEASE	—	PATIENT_INTRODUCTION/Disease/problem_history

^a^NICTA: National Information and Communications Technology Australia.

^b^Does not contain any matching label from the source domain.

For the source domain corpus, we used 1 domain-related dataset, which contained labels that were relevant but not exactly the same as in our target domain data, and 1 of the domain corpora, which contained many of the general labels, including some labels that were relevant to biometrics. With this setup, we were keen to find out whether the parameters learned from the same domain were more valuable than those from general English.

The related domain source corpus was the aforementioned I2B2. It included fully deidentified discharge summaries and progress notes from real hospital scenarios. All records had been manually annotated for concept, assertion, and relation information. The corpus contained entities of 7 different labels: PATIENT, DOCTOR, HOSPITAL, DATE, PHONE, LOCATION, and ID. They were potentially relevant to labels in our NICTA dataset.

The general domain source corpus was Bolt, Beranek and Newman (BBN), which has a 1 million-word Penn Treebank corpus of Wall Street Journal texts annotated by BBN with 28 main types of entities: 12 named entity types (Person, Facility, Organization, geographical entities (GPE), Location, Nationality, Product, Event, Work of Art, Law, Language, and Contact-Info), 9 nominal entity types (Person, Facility, Organization, GPE, Product, Plant, Animal, Substance, and Disease and Game), and 7 numeric types (Date, Time, Percent, Money, Quantity, Ordinal, and Cardinal). These types were further divided into 64 subtypes [46] (see Table 2 for the types related to labels in the target domain).

To examine what kind of word embeddings were most valuable to our task, we classified all the available datasets into 3 different groups: *Group 1 General (English Wikipedia+UMBC+One Billion)* was composed of general English materials, which do not contain many domain-specific words or sentences. *Group 2 Biomedical literature (PubMed+PMC)* was composed of biomedical literature and abstracts. Words in this group could be similar to clinical words but would be used in different ways, considering that publication writing is different from authoring clinical documents. *Group 3 Clinical documents (I2B2+NICTA Train)* was composed of clinical handovers, discharge summaries, and progress notes that closely resemble our task data. All corpora were preprocessed with the Stanford CoreNLP sentence splitter and tokenizer [47]. Digits were replaced with NUM[Length] (eg, 08-08-1988 is replaced by NUM2-NUM2-NUM4), this method helps to capture some digit patterns such as date and phone numbers and will dramatically decrease the amount of words in vocabularies as well. To compute vector representations of word, word2vec [48] was used and modified with an extra option to incrementally train word embeddings based on existing models being given new text materials. We inherited the best parameter settings for named entity recognition from a previous study [24] with 200-word vector dimensions, 5 words in the context window, 10 negative samples, start with a 0.05 learning rate, and run over 20 iterations.

Besides using word vectors as features, we also used a collection of hand-crafted features that were identical to our previous NICTA IE system [25] for performance tracking. For each feature of 1-word instance, a unigram with a window size of 3 *(w_{i-1}, w_i, w_{i+1})*, and bigrams with a window size of 2 *(w_{i-1}w_i, w_iw_{i+1})* were used. Features used in our experiments include the lemma, part of speech tag, and parse tree, top 5 candidates and top mapping retrieved from the Unified Medical Language System (UMLS) [49], medication score—derived from the Anatomical Therapeutic Chemical List, location, and frequency.

To track the performance improvement on this task, the following 10 baselines were included for comparison, they are: 1) *Benchmark*, 2) *TUC-MI-A*, 3) *TUC-MI-B,* 4) *ECNU_ICA-A,* 5) *ECNU_ICA-B,* 6) *LQRZ-A,* 7) *LQRZ-B,* 8) *Unigram NN,* 9) *Random,* 10) *Majority*.

#### Benchmark

This was the initial NICTA benchmark system on this task using a single-layer linear-chain CRF [50] with L2 regulator with the handcrafted features mentioned before as input. A detailed description of this system can be found in the study by Suominen et al [4].

Participants of Conference and Labs of the Evaluation Forum (CLEF) eHealth Evaluation Lab 2016 Task 1: The CLEF eHealth 2016 Task 1 required the participants to implement systems that are able to identify relevant text snippets from free-text nursing handovers [51]. Participants were expected to train their systems using the given training set, optimize their performances using the validation set, and their final result was tested on a previous confidential test set. It should be noted that the benchmark NICTA IE system was provided to participants in the CLEF task as well as feature generators and intermediate processing results [51]. Participants could start their experiments from any point based on our previous work with very little effort. In fact, all systems except a and b were started from the NICTA benchmark IE system.

- *TUC-MI-A* was based on our benchmark system; rather than using our default features, this method constructed a 41-feature set based on Stanford CoreNLP, latent Dirichlet allocation, regular expressions, and the ontologies of WordNet and UMLS features [10].
- *TUC-MI-B* optimized TUC-MI-A; 19 features were selected from the whole feature set with forward and backward greedy search.
- *ECNU_ICA-A* was a rule-based IE system to recognize bed number, room number, age, and doctor’s name and was combined with CRF results using the same feature collection with the organizers’ benchmark system [52].
- *ECNU_ICA-B* has the same system architecture as ECNU_ICA-A, except for CRF training, and a subcollection of features was used for different label types [52].
- *LQRZ-A* was a feed-forward neural network with one hidden layer initialized with uniform distribution. Inputs to this NN model are pretrained word embeddings from GoogleNews. No handcrafted features were used in this model [53].
- *LQRZ-B* firstly used a random forest to predict a subset of the tags and the previous NN to further discriminate between the remaining labels [53].

#### Unigram NN

The unigram NN was an implementation of a 2-layer, first-order linear-chain graph transformer [21] with handcrafted features weighted by word vectors as the first layer and a linear-chain CRF on top of it. The model was trained using AdaGrad. This is a baseline to show separately, from the multilayer NN, what is the performance gain from using word embeddings and transfer learning.

#### Other Baselines

We evaluated the task difficulty of labeling each word with 1 out of 37 classes by comparing 2 baseline systems: First, we built a system that assigns classes randomly. Second, we implemented another system that always predicts the majority class (ie, the most common class in the training set): *Random* to randomly select 1 class label for each instance and *Majority* to assign the majority class of Future_Goal/ TaskToBeCompleted/ExpectedOutcome for every instance.

## Results

Researchers worldwide have contributed to achieve a significant improvement on the clinical handover task because of a shared computational task organized in 2016 [51]. In this study, we have reported the results from our experiments on the test set (Table 3) and have also taken this opportunity to overview performance improvements in the task, to summarize methods that have been used to solve the problems so far, and to inspire researchers to work further on this task. Overall, the state-of-the-art benchmark has been increased from 38.2% to 41.6% F1 (*P*<.001; Wilcoxon text [54]). Our transfer learning method using BBN as source domain (Trans_BBN) outperforms all other methods.

**Table 3:** Results of transfer learning compared with baseline systems.

Method	MacPrec^a^	MacRec^b^	MacF1^c^	MicPrec^d^	MicRec^e^	MicF1^f^
Trans_BBN	*0.498* ^g^	*0.419*	*0.416*	0.547	*0.488*	0.516
Trans_I2B2	0.481	0.390	0.392	0.565	0.471	0.514
TUC-MI-B	0.493	0.369	0.382	0.500	0.505	0.503
ECNU_ICA-A	0.493	0.406	0.374	0.510	0.522	0.516
General+I2B2+train	0.477	0.361	0.354	*0.612*	0.483	*0.540*
I2B2+train	0.443	0.367	0.354	0.604	0.484	0.537
General	0.429	0.356	0.345	0.606	0.478	0.535
LQRZ-B	0.425	0.383	0.345	0.490	0.517	0.503
General+PubMed+PMC	0.409	0.346	0.334	0.606	0.474	0.532
Unigram	0.393	0.292	0.311	0.574	0.448	0.503
TUC-MI-A	0.423	0.300	0.311	0.503	0.443	0.471
LQRZ-A	0.411	0.307	0.308	0.563	0.472	0.514
ECNU_ICA-B	0.428	0.292	0.297	0.581	0.459	0.513
National Information and Communications Technology Australia	0.435	0.233	0.246	0.433	0.368	0.398
Random	0.018	0.028	0.019	0.018	0.030	0.022
Majority	0.000	0.029	0.001	0.016	0.027	0.020

^a^Macro averaged precision.

^b^Macro averaged recall.

^c^Macro averaged F1.

^d^Micro averaged precision.

^e^Micro averaged recall.

^f^Micro averaged F1.

^g^Italics indicate the best result over the column.

Transfer learning with I2B2 as a source model (Trans_I2B2) is also able to increase the overall macro F1 by 4.7% (*P*<.001) compared with models using a 2-layer NN with general word embeddings (General). When using the same collection of handcrafted features, a 2-layer NN model (Unigram) performs 6.5% better than a single-layer linear-chain CRF (NICTA). The same model (Unigram) gains 3.4% improvement of macro F1 (*P*<.001) by using word embeddings pretrained with a large text collection with general English (Wiki). Word embeddings trained with a domain-related corpus but different context (Wiki+PubMed+PMC) actually harm rather than help the result. This is possibly because although the domain-related corpus contains medical terms, which are also used in a clinical health care environment, the context of these terms is still very different from clinical handovers. On the contrary, documents used in a similar scenario (I2B2+train) show their advantage at this point. Finally, embeddings trained with a combination of I2B2+train with general English (Wiki_I2B2+train) do not help the system to increase the macro F1, but they yield the best result on micro F1.

## Discussion

### Principal Findings

It can be seen from the experiment results that the DL system using pretrained word representations as the input, and the proposed transfer learning technique, is able to achieve better performance.

When comparing the results of different system setups on different subclasses, we observed that word representations learned from different domains and the knowledge transferred from various sources affect the clinical IE system on certain subclasses.

Comparing with the best result of feature engineering methods used in TUC-MI [9], our transfer learning method performs 3.4% better without a labor-costing feature-selection procedure. Furthermore, in contrast with the rule-based methods used in ECNU_ICA [52], which require domain-specific experts to inspect data carefully and make the rules, our method is much more efficient and still able to achieve a 4.2% better macro F1. Finally, the best LQRZ [53] used a very similar architecture with our General model, and we can see their performance is very similar as well; the minor difference is caused by different materials to train the word embeddings. Our transfer learning method is able to improve 7.1% macro F1 on top of the General model (*P*<.001).

In this section, we have analyzed these results and discussed these effects. The default measure will be the official macro F1 unless specifically mentioned otherwise.

### Word Representations

Word embeddings trained from general English can improve the clinical IE performance. Our results show that the *General* model, which used exactly the same model structure and feature map as the *Unigram* model, except it used a combination of 3 large corpora (English Wiki, UMBC, and One Billion) to train general word embeddings, performed better on the overall task (34.5% vs 31.1%, respectively; *P*<.001). This indicates that word representations trained from unlabeled general English text are able to capture word features that contribute to classifying different annotations in clinical handovers.

Moreover, general word embeddings fine-tuned with a small task-relevant dataset can further increase the result. The model trained with I2B2 and NICTA training data *(I2B2+train)* outperforms the *Unigram* model by 4.3% and outperforms general embeddings when it is compared with the *General* model (35.4% vs 34.5%, respectively; *P*=.17).

However, no evidence was found to indicate that continuing training word embeddings with a relevant dataset based on pretrained general word embeddings contributes to the system performance when comparing the *I2B2+train* with *General+I2B2+train*. This might be because although the corpora of I2B2 and NICTA training data are significantly smaller than the general English corpus, the vocabulary is still enough to cover words that are present in the test set, and after several iterations of training, word embeddings in these 2 different settings eventually converged to similar values.

Word embeddings trained from domain relevant data do not show any evidence to contribute to improving the system result either. Our results showed that the *General+PubMed+PMC* model performed worse than the *General* model (33.4% vs 34.5%, respectively; *P*=.07). This might be because even though we considered clinical and biomedical areas as relevant, but because of having different scenarios, vocabulary and context could end up too different. This would introduce more noise to the word embeddings and so does not contribute to the IE performance.

### Transfer Learning

Transfer learning shows its advantage in the clinical handover IE task. The top 2 systems were both transfer learning models. Transfer learning from BBN *(Trans_BBN)* was 3.4% higher than the previous best system *TUC-MI-B* (41.6% vs 38.2%, respectively; *P*<.001).

For the overall result, there is no strong evidence to show any advantage of transfer from domain-relevant source data *(Trans_I2B2)* over general annotations *(Trans_BBN)*. On the contrary, transfer learning from BBN with general annotations performed slightly better than I2B2, which contains more relevant entities with our target task on macro F1 (41.6% vs 39.2%, respectively; *P*<.001).

For subclasses, Table 4 shows the results of transfer learning compared with the baseline system when the performance is improved on subclasses. When referring to Table 2:

1. Some subclasses where the performance is improved by transfer learning *HAVE* a mapping annotation type from the source domain: for example, subclass PATIENT_INTRODUCTION: Age in years has a mapping annotation DATE:AGE in the source domain BBN, and Trans_BBN on this subclass performed better than the General model (96.5% vs 94.8%, respectively; *P*<.001). This indicates that when the target domain labels have mappings from the source domain annotations, transfer learning can improve the extraction results of these labels.
2. Some subclasses where the performance is improved by transfer learning *do not have* a mapping annotation type from the source domain: for subclass FUTURE_CARE: Alert/waring/abnormal result, the general model was not able to predict any instance correctly, whereas transfer learning did learn some knowledge from the training set but the performance was still not very high. This might be because these subclasses may have some underlying correlations with source domain labels that are automatically learned during the second process in our method, even though the correlations were not straightforward or obvious for human readers.
3. Some subclasses that have mappings from the source domains do not gain any improvement from transfer learning: for example, PATIENT_INTRODUCTION/ Given_names. These classes normally already have good performance from only using general models, so transfer learning, in this case, might introduce extra noise from other domains that potentially have different sentence structures to the target domain, and thus harm the results.

**Table 4:** Results of subclasses when transfer learning improved in the baseline system (F1 score).

Entity type	Instances (n)	General	Trans_I2B2	Trans_BBN
PATIENT_INTRODUCTION: Age (years)	246	0.948	0.879	*0.965* ^a^
PATIENT_INTRODUCTION: Gender	88	0.826	0.896	*0.917*
PATIENT_INTRODUCTION: Admission reason	412	0.214	0.311	*0.344*
PATIENT_INTRODUCTION: Chronic condition	70	0.000	*0.105*	0.081
PATIENT_INTRODUCTION: Disease/problem history	147	0.016	*0.083*	0.044
PATIENT_INTRODUCTION: Care plan	36	0.069	0.129	*0.133*
PATIENT_INTRODUCTION: Allergy	14	0.267	0.500	*0.566*
APPOINTMENTS: Time	28	0.114	*0.431*	0.400
APPOINTMENTS: Place: Ward	3	0.000	0.000	*0.400*
APPOINTMENTS: Status	159	0.111	*0.175*	0.132
FUTURE_CARE: Alert/warning/abnormal result	59	0.000	0.087	*0.178*
FUTURE_CARE: Goal/task to be completed/expected outcome	496	0.000	0.068	*0.070*
FUTURE_CARE: Discharge/transfer place	89	0.327	0.288	*0.361*
MY_SHIFT: Status	481	0.570	*0.688*	0.638
MY_SHIFT: Input/diet	101	0.413	0.783	*0.804*
MY_SHIFT: Output/diuresis/bowel movement	52	0.286	0.396	*0.478*
MY_SHIFT: Wounds/skin	55	0.444	0.357	*0.457*
MY_SHIFT: Activities of daily living	245	0.579	*0.753*	0.748
MY_SHIFT: Other observation	361	0.177	*0.220*	0.202
MEDICATION: Medicine	156	0.450	*0.548*	0.495
MEDICATION: Status	68	0.034	*0.086*	0.085

^a^Italics indicate the best result over the column.

### Conclusions

This study investigated adapting a DL method to extract patient information from clinical reports. Domain and task specification word representations have been used as inputs to a DL system to achieve better performance. In addition, a transfer learning model has been applied to adapt knowledge learned from general text sources to a domain-specific task. This method was able to further improve the overall result, especially in the classes related to the source domain. Domain-specific word representations improve the overall clinical IE system performance by 3.4% on macro-F1. Transferring the knowledge from a general English corpus to our task-specific domain gains a further 7.1% improvement. To our knowledge, our study is the first attempt to transfer knowledge from general deep models to specific tasks in health care and gain a significant improvement. The result of our system is state-of-the-art on this task. Our method and result point out the way toward adapting an advanced ML technique to professional informatics system tasks.

## Appendix

Multimedia Appendix 1 Dataset description.

Multimedia Appendix 2 The test set of our experiments in the format of each row contains the word with its features, and the last column is the human-assigned label.

## Abbreviations

AdaGrad: adaptive gradient algorithm
BBN: Bolt, Beranek and Newman
CLEF: Conference and Labs of the Evaluation Forum
CRF: conditional random field
DL: deep learning
GPE: geographical entities
IE: information extraction
ML: machine learning
NICTA: National Information and Communications Technology Australia
NN: neural network
NLM: National Library of Medicine
NLP: natural language processing
PMC: PubMed Central
RN: registered nurse
SR: speech recognition
UMBC: University of Maryland, Baltimore County
UMLS: Unified Medical Language System
