# Supplementary material for: Adapting State-of-the-Art Deep Language Models to Clinical Information Extraction Systems: Potentials, Challenges, and Solutions
Source: JMIR Med Inform. 2019 Apr 25;7(2):e11499. doi: 10.2196/11499 (PMC6658232; doi:10.2196/11499)
Supplement: Multimedia Appendix 1 [file medinform_v7i2e11499_app1.pdf]

NICTA Synthetic Nursing Handover Data

The data set is called **NICTA Synthetic Nursing Handover Data.** It has been developed for clinical speech recognition (SR) and information extraction (IE) related to nursing shift-change handover at NICTA/Data61 from 2012 to 2016. The dataset is documented in Suominen H, Zhou L, Hanlen L, Ferraro G. Benchmarking clinical speech recognition and information extraction: New data, methods and evaluations. *JMIR Medical Informatics* 2015 3(2), e19. (PDF) .

The data set has been created by Maricel Angel, Registered Nurse (RN) with over 12 years' experience in clinical nursing. The text is thus very similar to real documents in Australian English (which cannot be made available). This data creation included the following steps:

1. generation of **patient profiles**,
2. creation of **written, free-form text documents**,
3. development of a **structured handover form with 50 headings to fill out**,
4. using this form and the written, free-form text documents to create **written, structured documents**,
5. creation of **spoken, free-form text documents**,
6. using a **speech recognition engine** with different vocabularies to convert the spoken documents to written, free-form text, and using an **information extraction system** to fill out the handover form from the written, free-form text documents.

The data release has been approved and the RN has been consented in writing. The license of the spoken, free-form text documents (i.e., WMA and WAV files) is **Creative Commons - Attribution Alone - Non-commercial - No Derivative Works (CC-BY-NC-ND)** for the purposes of testing speech recognition and natural language processing algorithms. The remaining documents (i.e., DOCX and TXT files) are licensed under **Creative Commons - Attribution Alone (CC-BY)**.

For your convenience, we are offering you two alternatives to access the mutually exclusive **training data** (called **data set 1 with 101 synthetic patient cases**), **validation data** (called **data set 2 with another 100 synthetic patient cases**), and **test data** (called **data set 3 with yet another 100 synthetic patient cases**) as follows:

Option 1: Through CSIRO Data Access Portal **here** (supplemented by the audio files for SR).

- Training set: go to Files —> data set 1 - text files —> 101writtenfreetextreports and 101informationextraction for the free-form text documents and their gold standard annotations, respectively.
- Validation set: go to Files —> data set 2 - text files —> 100writtenfreetextreports and 100informationextraction for the free-form text documents and their gold standard annotations, respectively.
- Test set: See Option 2, Test set below.

Option 2: Through Google Drive.

- Training set: data set 1 .
- Validation set: data set 2.
- Test set: **data set 3** . For your convenience, its key elements are also available in the CSV format on Kaggle and in **here**.

Participants must not use the test set for development. The test set is divided in halves (50%) between the public and private leaderboard on Kaggle. However, repeated testing on test data is likely to lead to over-fitting your solution.

Using authentic or synthetic clinical data sets other that these data sets 1 and 2 for setting up the system is **not permitted**. However, using **standard data sets, even with annotations, (e.g., Clueweb, LDC Databases, and Wikipedia),** for example, to utilise word embedding or transfer learning is permitted, but this needs to be carefully documented in the submission description.
